# Development of six PROMIS pediatrics proxy-report item banks

**DOI:** 10.1186/1477-7525-10-22

**Published:** 2012-02-22

**Authors:** Debra E Irwin, Heather E Gross, Brian D Stucky, David Thissen, Esi Morgan DeWitt, Jin Shei Lai, Dagmar Amtmann, Leyla Khastou, James W Varni, Darren A DeWalt

**Affiliations:** 1 Department of Epidemiology, University of North Carolina at Chapel Hill, CB #7295 Chapel Hill, NC, USA 27599; 2 Department of Psychology, University of North Carolina at Chapel Hill, Chapel Hill, NC, USA; 3 Department of Pediatrics, Division of Rheumatology, James M. Anderson Center for Health Systems Excellence, Cincinnati children's Hospital and Medical Center, Cincinnati, OH, USA; 4 Department of Medical Social Sciences, Northwestern University Feinberg School of Medicine, Chicago, IL, USA; 5 Center on Outcomes Research in Rehabilitation, University of Washington, Seattle, WA, USA; 6 Department of Pediatrics, College of Medicine, Department of Landscape Architecture and Urban Planning, College of Architecture, Texas A&M University, College Station, Texas, USA; 7 Division of General Medicine and Clinical Epidemiology and the Cecil G. Sheps Center for Health Services Research, University of North Carolina at Chapel Hill, Chapel Hill, North Carolina, USA; 8 Cecil G. Sheps Center for Health Services Research, University of North Carolina at Chapel Hill, Chapel Hill, North Carolina, USA

**Keywords:** PROMIS, HRQOL, PRO, Scale development, Parent Proxy, Pediatrics

## Abstract

**Background:**

Pediatric self-report should be considered the standard for measuring patient reported outcomes (PRO) among children. However, circumstances exist when the child is too young, cognitively impaired, or too ill to complete a PRO instrument and a proxy-report is needed. This paper describes the development process including the proxy cognitive interviews and large-field-test survey methods and sample characteristics employed to produce item parameters for the Patient Reported Outcomes Measurement Information System (PROMIS) pediatric proxy-report item banks.

**Methods:**

The PROMIS pediatric self-report items were converted into proxy-report items before undergoing cognitive interviews. These items covered six domains (physical function, emotional distress, social peer relationships, fatigue, pain interference, and asthma impact). Caregivers (n = 25) of children ages of 5 and 17 years provided qualitative feedback on proxy-report items to assess any major issues with these items. From May 2008 to March 2009, the large-scale survey enrolled children ages 8-17 years to complete the self-report version and caregivers to complete the proxy-report version of the survey (n = 1548 dyads). Caregivers of children ages 5 to 7 years completed the proxy report survey (n = 432). In addition, caregivers completed other proxy instruments, PedsQL™ 4.0 Generic Core Scales Parent Proxy-Report version, PedsQL™ Asthma Module Parent Proxy-Report version, and KIDSCREEN Parent-Proxy-52.

**Results:**

Item content was well understood by proxies and did not require item revisions but some proxies clearly noted that determining an answer on behalf of their child was difficult for some items. Dyads and caregivers of children ages 5-17 years old were enrolled in the large-scale testing. The majority were female (85%), married (70%), Caucasian (64%) and had at least a high school education (94%). Approximately 50% had children with a chronic health condition, primarily asthma, which was diagnosed or treated within 6 months prior to the

interview. The PROMIS proxy sample scored similar or better on the other proxy instruments compared to normative samples.

**Conclusions:**

The initial calibration data was provided by a diverse set of caregivers of children with a variety of common chronic illnesses and racial/ethnic backgrounds. The PROMIS pediatric proxy-report item banks include physical function (mobility n = 23; upper extremity n = 29), emotional distress (anxiety n = 15; depressive symptoms n = 14; anger n = 5), social peer relationships (n = 15), fatigue (n = 34), pain interference (n = 13), and asthma impact (n = 17).

## Background

The Patient Reported Outcomes Measurement Information System (PROMIS) project, a National Institutes of Health initiative, was developed to advance the science and application of patient-reported outcomes (PRO) [1]. One main goal of the PROMIS initiative was to develop a set of item banks and computerized adaptive tests for the clinical research community. The PROMIS pediatric project focused on the development of self-report PRO item banks across several health domains for youth ages 8-17 years. The primary focus was on the measurement of generic health domains that are important across a variety of health states (including physical function, pain interference, fatigue, emotional distress, and social peer relationships) [2-8]. Additionally, one disease specific item bank was developed for children with asthma to explore the relationships between general and disease specific measures [9].

It is well documented in both the adult and pediatric literature that information provided by proxy respondents is not equivalent to that reported by the patient [10-14]. Imperfect agreement between self-report and proxy-report, termed cross-informant variance [15], has been consistently documented in the health related quality of life (HRQOL) measurement of children with chronic health conditions and healthy children [12,16]. Consistencies between child and parent proxy-reports as measured by intra-class correlation coefficients have been reported as low as 0.02 to 0.23 [13]. However, even as pediatric patient self-report is advocated, there remains a role for parent proxy-report in pediatric clinical trials and health services research.

Although pediatric patient self-report should be considered the standard for measuring PROs, there are often circumstances when the child is too young, cognitively impaired, too ill, or fatigued to complete a PRO instrument and a parent proxy-report may be needed [17]. Further, it is typically parents' perceptions of their child's symptoms and outcomes that influence healthcare utilization [17-19]. Optimally, PRO instruments should be selected that measure the perspectives of both the child and the parent since these perspectives may be independently related to healthcare utilization, risk factors, and quality of care [20]. Hence, the PROMIS pediatric project undertook the development of proxy item banks across several health domains (physical function, pain interference, fatigue, emotional distress, social peer relationships and asthma impact) for youth ages 5-17 years.

This process of developing item banks for PROMIS included literature review, qualitative research including individual cognitive interviews and field-testing [2,4,21-23]. This paper describes the development process employed for the PROMIS Pediatric Parent Proxy item banks. Specifically, the proxy cognitive interview process and results as well as the methods utilized and the final sample characteristics of the large-scale field-test survey designed to produce item parameters are reported. Other manuscripts will describe in detail the psychometric properties of the proxy item banks administered during large-scale field-testing [24].

## Methods

### Proxy item bank development

PROMIS domain definitions were previously published [4] and cover six domains (physical function which includes mobility and upper extremity, emotional distress which comprises of anger, anxiety and depressive symptoms, peer relationships, fatigue, pain interference, and asthma impact). The proxy-report items were developed from the existing PROMIS pediatric self-report content domains [3-7,9] and aimed to include proxy respondents with children ages 5-17 years of age. The items were revised to retain their meaning, while modifying the phrasing so that all items involved parents/caregivers reporting on their 5-17 year old children. For example, in the PROMIS pediatric self-report pain interference instrument [5], children responded to the item "I had trouble sleeping when I had pain," while caregivers responded to the proxy-report equivalent of this item, "My child had trouble sleeping when he/she had pain."

All items had a 7-day recall period and used standardized 5-point response options *(e.g., never, almost never, sometimes, often, almost always; or, with no trouble, with a little trouble, with some trouble, with a lot of trouble, not able to do) *or a numeric rating from 0- 10. The pediatric self-report items were the basis for the proxy-report items as they were developed utilizing a process that reflected the language and context important to children [4]. All of the pediatric self-report items were converted into proxy-report items and new items were not created. The expert item development team (n = 8) which included pediatricians, psychometricians, epidemiologists, pediatric psychologists as well as survey development experts, felt in their experience most researchers would not want a proxy-report item set that was different from the child self-report. The team recognized that this was a decision based on empirical experience and has not been documented in the published literature.

One of the initial components of the PROMIS child-report item bank development process was soliciting input on the potential PROMIS items through focus groups with children and caregivers [22]. In addition, the PROMIS child self-report items all underwent extensive cognitive interviewing with children [23]. Hence, it was not anticipated that major item revisions would be needed for the adaption of the child self-report items to a proxy-report format. One of the key issues that the proxy cognitive interviews addressed was the understandability and confidence of proxy report on the various PROMIS domains.

A total of 185 items were sampled from the initial PROMIS pediatric item banks (n = 293 items) and underwent cognitive interviews. These items represented all content areas within the domains and were chosen by the expert item development team based on which items thought most likely to exhibit issues with proxy reporting. The primary purpose of the proxy cognitive interviews was to derive qualitative information on whether there were major issues with the converted proxy-report items and to provide insight into which items or domains proxy respondents felt most comfortable answering. A complete list of proxy-report items is available elsewhere [24].

### Proxy cognitive interviews

#### Recruitment and participants

To participate in the proxy cognitive interviews, participants needed to be the primary caregiver (parent or guardian) of a child between the ages of 5 and 17 years inclusive, speak and read English, and provide informed consent prior to study entry. We also specifically recruited caregivers of children with asthma to review all asthma-specific items.

A research assistant (RA) approached caregivers of children who appeared to be between the ages of 5 and 17 years old and who were waiting for their child's clinic appointment at the University of North Carolina's (UNC) general and subspecialty pediatrics clinics. In addition, a study recruitment email was sent through the general UNC employee and student email system to recruit caregivers from a non-clinic population. The RA provided an explanation of the study and scheduled an interview appointment was scheduled for eligible participants. At the time of the interview, a trained RA obtained informed consent and administered the interview. Participants received a $25 gift card in return for their time and effort. The study protocol was approved by the institutional review board. Cognitive interviews were conducted from November 2007 through January 2008.

A total of 25 parents were recruited to participate in the cognitive interviews. For each item, the cognitive interview sample included at least 5 caregivers - 2 caregivers of children of non-white ethnic/racial background and 3 caregivers of children ages 5-7 years old. These categories were not exclusive. For example, a parent of a Hispanic girl age 6 would fulfill both the racial/ethnic requirement and the age requirement. The first 25 caregivers who met these sampling criteria were interviewed. Caregivers with children ages 5-7 were purposely oversampled to ensure that the item content was appropriate for proxy respondents with children in this age groups. Our earlier published work verified that the content was suitable for children as young as 8 years old [23].

#### Proxy cognitive interview process

We applied a sampling scheme that allowed each participant to be interviewed for approximately 1 h on approximately 30 to 40 items rather than all 185 items. By this method, the vast majority of the items in the bank were reviewed by at least 5 participants (97%) meeting the target demographic characteristics outlined above (see Recruitment and Participants Section). Caregivers with asthmatic children underwent the cognitive interview on the asthma-specific item set while the other participants were randomly assigned to receive another item set. During the cognitive interviews, participants were asked to provide verbal open-ended feedback on each item regarding response categories, time frame, item interpretation and overall impression of domain content and coverage. These questions (see Proxy Cognitive Interview Questions section) were based on prior published work [23] and developed by the expert item development team.

#### Proxy cognitive interview questions

##### Items

How would you say this question in your own words? How easy or hard was this question to answer? (If difficult to answer) How would you change the words to make it easier to answer?

##### Directions

When you answered the question, what time frame were you considering? When you answered the question, did you think only about the past 7 days or did you need to think farther back in time? Or did you consider the past couple of days?

##### Overall Assessment

You may have noticed that many of our questions contain the words he/she, him/her or himself/herself. As you hear these questions, did you find this kind of wording to be awkward? If so, is there another way that you would suggest we word these questions? In general, how easy or difficult was it to answer these questions about your child? Explain. Are there things that we forgot to ask about that you think are important? Overall thoughts/opinions of the questionnaire?

Prior to the cognitive interview, participants completed an item set through paper and pencil administration. Caregivers were also asked to complete a sociodemographic form to report information regarding the child's age, gender, ethnicity, race, and chronic health condition(s) as well as the parent/guardian's marital status, employment status, and educational level (Table 1).

**Table 1:** Proxy Caregiver's Demographics and Clinical Characteristics for Cognitive Interviews

	N = 25 (%)
***Child's Gender-***Female	13 (52)

***Child's Age***	
5-7 years	17 (68)
8-9 years	3 (12)
10-12 years	0 (0)5
13-17 years	(20)

***Child's Grade Completed in School***	
1^st ^or less	17 (68)
2^nd^-5^th^	3 (12)
6^th^-11^ths^	5 (29)

***Child's Race***	
Caucasian	14 (56)
African American	10 (40)
Other-Mixed	1 (4)

***Child's Ethnicity-***Hispanic	2 (8)

***Caregiver's Marital Status***	
Divorced/Separated	6 (24)
Married	13 (52)
Never married	6 (24)

***Caregiver's Education Status***	
Advanced degree	10 (40)
College	5 (20)
Some college/AA	5 (20)
High School	3 (12)
Some high school	2 (8)

***Caregiver's Occupation***	
Full-Time Employed	12 (48)
Part Time Employed	7 (28)
Not employed	6 (24)

Chronic Health Condition in Past 6 Months*	17 (68)

***Asthma***	
Child ever diagnosed with asthma and currently treated	14 (56)

*caregivers reported child was diagnosed or treated for a chronic health condition within 6 months prior to the interview and these conditions included (asthma, migraines, anxiety, ADHD/ADD, and allergies)

All research assistants performing the cognitive interviews underwent 8 h standardized cognitive interview technique training and had extensive experience conducting cognitive interviews prior to this study. These interviewers reviewed each item stem and item response with the caregiver and began the interview using standardized questions (see Proxy Cognitive Interview Questions section) for each item.

#### Proxy cognitive interview data analyses

The cognitive interviews were audiotaped to allow for recording of detailed participant responses. After completing the initial cognitive interviews for an item, the research assistants conducting these interviews utilized the audio tapes to transcribe and compile a summary statement for each item including the caregiver's quotes and comments for each cognitive interview question. The expert item development team then reviewed all of the summary statements from each respondent for each item to determine issues with item comprehension, instructions, relevance and other general issues. Cognitive interview results were discussed for every item and a consensus decision was made as to the disposition of each item.

#### Proxy field-test survey instrument

Following the expert item development team review of all cognitive interview results, the PROMIS proxy items were assembled in a survey to be utilized in large-scale data collection [24]. The sampling plan for the child participants is described in detail elsewhere [4]. The caregiver proxy forms contained PROMIS proxy items and items from widely used fixed length measures including the PedsQL™ 4.0 Generic Core Scales Parent Proxy-Report version [17], PedsQL™ Asthma Module Parent Proxy-Report version [25], and KIDSCREEN Parent-Proxy-52 [26-28]. These instruments are scored on a 0-100 scale, with higher scores indicating better HRQOL [17,25-28]. The PedsQL™ 4.0 Generic Core Scales Parent Proxy-Report version yielded scale scores for physical functioning, emotional functioning, social functioning, school functioning, psychosocial health summary score and total summary score; the PedsQL™ Asthma Module Parent Proxy-Report version yielded scale scores for asthma symptoms, treatment, worry and communications; and the KIDSCREEN Parent-Proxy-52 yielded scale scores for physical well-being, psychological well-being, moods and emotions, self-perception, autonomy, parent relations and home life, financial resources, social support and peers, school environment, and social acceptance and bullying. These measures were chosen because they are widely used in assessing pediatric quality of life and they were administered in our child large-scale survey [4]. For simplicity purposes, they will be referred to as legacy items or scales throughout this manuscript.

The 293 proxy-report items from 6 general domains (Physical Function, Pain Interference, Fatigue, Emotional Distress, Social Peer Relationships and Asthma Impact) were administered to 1980 caregivers of children participating in the study. Because Physical Function includes both upper extremity and mobility item banks, Emotional Distress includes separate anger, anxiety and depressive symptoms item banks, and Fatigue includes both fatigue and lack of energy item banks, a total of 10 content areas were tested. A complete list of final PROMIS proxy items is published elsewhere [24]. To reduce respondent burden, a multi-form design was used in which the items were divided among nine test forms (one for caregivers of children with asthma and eight for other caregivers), and each caregiver was administered one of the forms (Table 2). To ensure an adequate number of individuals responded to all items, each item appeared on three of the forms. This process resulted in all items being administered to at least 428 parents. Caregiver participants were sequentially assigned to complete one of the eight testing forms and caregivers of children diagnosed with asthma were specifically assigned to the form containing asthma items.

**Table 2:** Distribution of Items by Proxy Test Administration Form*

Item Banks (number of total items)	Form 1*	Form 2*	Form 3*	Form 4*	Form 5	Form 6	Form 7	Form 8	Form Asthma
PROMIS Emotional Distress-Anger(n = 10)	10 items			10 items	10 items				4 items*

PROMIS Emotional Distress- Anxiety(n = 18)	18 items	9 items		9 items	9 items	9 items			4 items*

PROMIS Emotional Distress-Depressive Symptoms (n = 21)	21 items	11 items		10 items	10 items	11 items			4 items*

PROMIS Fatigue (n = 39)	14 items	20 items	15 items	19 items	6 items	8 items	12 items	13 items	4 items*

PROMIS Pain (n = 27)	13 items	14 items	14 items	13 items	6 items	7 items	7 items	7 items	3 items*

PROMIS Physical Function-Mobility (n = 32)	13 items	17 items	19 items	15 items	6 items	7 items	10 items	9 items	4 items*

PROMIS Physical Function-Upper Extremity (n = 38)	14 items	20 items	24 items	18 items	6 items	8 items	12 items	12 items	4 items*

PROMIS Social Peer Relationships (n = 74)	26 items	38 items	48 items	36 items	12 items	14 items	24 items	24 items	8 items*

PROMIS Asthma Impact (n = 34)									34 items

Legacy Items					PedsQL Generic	PedsQL Generic Core	PedsQL Generic Core	PedsQL Generic Core	PedsQL Asthma Module 28 items;

					Core Scales 23 items; KIDSCREEN 52 items	Scales 23 items; KIDSCREEN 52 items	Scales 23 items; KIDSCREEN 52 items	Scales 23 items; KIDSCREEN 52 items	DISABKIDS Asthma Module 14 items

*some items are duplicated between forms

The caregivers survey also included questions to assess sociodemographic items including the child's age, sex, race, ethnicity, and education as well as the caregiver's marital status, education level, occupational status and the medical history of the child. The medical history included diagnoses of any new chronic health conditions within six months prior to study enrollment, treatment for existing chronic health conditions within six months prior to study enrollment, lifetime diagnosis of asthma, and current asthma treatments.

This sampling plan was developed for collecting responses to the candidate items from the targeted PROMIS domains and was designed to accommodate multiple objectives: (1) assess the factor structure of the domains, including tests of local dependence (LD); (2) evaluate items for differential item functioning (DIF); and (3) calibrate the items for each domain using item response theory (IRT).

#### Field-test recruitment and participants

The survey participants included a diverse group of caregiver (parent or guardian)-child dyads (for children ages 8-17 years old) as well as caregivers (parents or guardians) of children ages 5-7 years old. Children were selected to have diversity in gender, age, race/ethnicity groups, and health status (e.g., children with a variety of common chronic illnesses) in order to have a range of representation across the latent traits measured by the item banks.

To be eligible to participate in the large-scale testing survey, all participants were required to speak and read English and be able to see and interact with a computer screen, keyboard, and mouse. Children enrolled in the study were between the ages of 8 and 17 years and along with their parents or guardians formed a caregiver-child dyad. These caregiver-child dyads (children ages 8-17 years old) will simply be referred to as dyads. For children 8-17 years old, both members of the dyad were required to complete the survey during the same testing session. Children completed the pediatric self-report version of the survey and parents or guardians completed the proxy-report version of the survey. Parents or guardians (caregivers) of children between the ages of 5 and 7 years of age were also enrolled and only the caregiver completed the proxy report survey. The study sample also included dyads for children ages 8-17 years old or caregivers of children ages 5-7 years old diagnosed with asthma. The asthmatic children were required to be diagnosed by a physician prior to study participation and currently using asthma medication.

Participants were recruited in outpatient general pediatrics and subspecialty clinics. Potential clinic pediatric participants were identified through a variety of methods such as a review of pediatric clinic appointment rosters or while in the clinic waiting rooms according to protocols approved by the institutional review boards (IRBs) of the University of North Carolina (UNC), Duke University Medical Center, University of Washington Center on Outcomes Research in Rehabilitation (UW), children's Memorial Hospital (CMH) in Chicago, and The children's Hospital at Scott and White (S&W) in Texas. Pediatric patients within the appropriate age range who had clinic appointments and their caregivers were recruited while waiting for their clinic appointments. The UNC, Duke, UW, CMH and S&W general pediatric clinics see patients with a broad spectrum of health issues (e.g., well child visits, acute illnesses, and some chronic illnesses). The specialty clinics including Pulmonology, Allergy, Gastroenterology, Rheumatology, Nephrology, Obesity, Rehabilitation, Dermatology, and Endocrinology, primarily saw children with more serious chronic illnesses. Children with asthma and their caregivers were over sampled during recruitment because asthma-specific items were tested.

Caregivers signed an informed consent document and children signed an informed assent document that outlined the following: purpose of the study, participation requirements, potential benefits and risks of participation, and the measures implemented to protect participant privacy. The survey was administered on laptop computers in a private location. Children completed the survey at the time of recruitment without assistance. Each member of the dyad or the caregiver of children ages 5-7 years was assigned to complete one of the test administration forms (Table 2). Each participant received a $10 gift card in return for their time and effort. The study protocols were approved by the institutional review boards at each institution. Data were collected from May 2008 through March 2009.

### Field-test data analysis

Descriptive analyses were conducted to describe the demographic and clinical characteristics of the study sample. Mean scores for legacy scales were calculated per instrument instructions [17,25-28] in order to compare on a descriptive basis normative sample data for legacy instruments and the PROMIS proxy sample scores on these legacy instruments. Detailed psychometric properties of the proxy-report item banks are described in another manuscript [24].

## Results

### Proxy cognitive interviews

Table 1 shows the general characteristics of the children and caregivers (all participants were parents) who participated in the cognitive interviews. The interviews did not find any issues around item content or understanding that would require item revisions. This is not surprising considering that all the items had previously undergone cognitive interviews with children.

Proxy respondents mentioned issues related to response options and wording emphasis for two items ("*It was hard for my child to play with pets because of asthma" *and *"How many days did your child have no pain*"). One caregiver requested a 'not applicable' option be added to the response options for the asthma item and that the word 'no' be emphasized in the pain item. Neither of these changes was made because it was decided that it was more important to keep the proxy-report items and the self-report items similar as most researchers do not want different proxy and child-report item sets.

A common theme that emerged from the cognitive interviews was that 14 out of 25 parents expressed difficulty in answering some of the items because they did not know enough information to reliably report for their child (Table 3). The most common reasons given were that the child did not share the information with them or that they did not observe the child in enough settings to adequately assess the information. This occurred across several domains but most predominately for the social peer relationship domain. In all cases, the parents answered the corresponding item on the paper/pencil version of the questionnaire despite expressing their difficulty during the cognitive interview portion of the study.

**Table 3:** Items Caregivers Reported Feeling Uncomfortable Answering

Item	Number of Caregivers Expressing Difficulty Answering Item/Number of Caregivers Answering Item	Selected Caregiver Quotes
***Asthma Impact Items***		

My child's chest felt tight because of asthma.	2/5	"...she has never said anything directly to me so I had to guess"

My child got tired easily because of his/her asthma.	1/5	" I don't see her at school when she would be running around"

My child's body felt bad when he/she was out of breath.	1/5	" I don't know how his body felt, son didn't tell me how his body felt."

***Emotional Distress Items***		

My child worried about what could happen to him/her.	2/5	"My child doesn't always share his emotions with me."

My child got scared really easily.	1/5	"It is harder to ascertain my child's feelings"

My child felt alone.	1/5	"This was hard to answer because if she didn't tell me I wouldn't know."

My child was worried when he/she was away from home.	2/5	"It's not so much the wording it's that you're asking me to report something that I don't know"

Being sad made it hard for my child to do things with friends.	1/5	"I couldn't relate to questions about my child's feelings because I have no idea."

***Fatigue Items***		

My child was so tired it was hard for him/her to pay attention.	1/5	" I just answered to the best I could. I'm not always there when she would need to pay attention like at school"

***Pain Items***		

It was hard for my child to think when he/she had pain.	2/5	"I cannot measure/gage if he can think when he has pain as he may not tell me"

It was hard for my child to remember things when he/she had pain.	1/5	"I don't know what he's thinking when he has pain."

***Social Role Relationship Items***		

My child was good at making friends.	2/5	"The friends piece comes at school and I'm not there"

It was hard for my child to make friends.	2/5	"I have no idea about things that occur at school or after- school that I can't see. These are questions about things that I haven't seen with my own eyes."

Other kids teased my child.	1/5	"I'm not at school to see this"

Other kids did not want to be my child's friend.	2/5	"These are things that are internal that I can't see as a parent"

Other kids bullied my child.	1/5	"... if he doesn't talk about it or I don't see it happen... then I don't know""

My child was good at making friends.	2/5	"Anything that I'm able to observe is a lot easier to answer (vs. questions about how my child feels)."

My child felt accepted by other kids his/her age.	1/5	"I don't have a gauge to know how he felt. I have to guess"

Other kids did not want to be my child's friends.	2/5	"Some questions were harder due to difficulty knowing how to answer on behalf of child's experience "

My child was able to have fun with his/her friends.	1/5	"this question was a little harder-I had to think about whether she had fun since I'm not always with her"

My child was able to count on his/her friends.	1/5	"I had to think again about what she has told me."

My child treated other kids his/her age with respect.	1/5	"This was a little harder to answer- because I'm not around her when she's around other kids."

Other kids did not like my child.	1/5	"This was harder to answer because I am not around my child at school."

My child felt nervous when he/she was with other kids the same age.	2/5	"I'm not sure what she is like when she is around other kids."

Other kids wanted to be with my child.	2/5	"This is hard to answer because I'm not around her during the school day."

Other kids wanted to be my child's friend	1/5	"With an older teenager it's a little more difficult to know."

Other kids were mean to my child.	1/5	"It depends on whether the child tells the parents or if I observed it directly."

My child teased other kids.	1/5	"It was impossible to know about other kids since I wasn't there."

My child played alone and kept to himself/herself.	1/5	"I thought of it as "at our house". Because in school, Sunday school, etc. I do not have the foggiest idea."

### Large-scale proxy testing

Dyads (n = 1548) and caregivers of 5-7 year olds (n = 432) were enrolled and the sample characteristics were similar between both groups (Table 4). The majority of caregivers were female (85%), married (69% for children ages 8-17; 71% for children 5-7 years old), Caucasian (64%) and had at least a high school education (94%). Approximately 50% had children with a chronic health condition diagnosed or treated within 6 months prior to the interview which was primarily asthma (23% for children ages 8-17; 26% for children ages 5- 7).

**Table 4:** Caregivers and Child Characteristics for Proxy Large-scale Survey

	N = 1548 dyad for children ages 8-17 years (% of complete data)	N = 432 for caregivers of children 5 7 years old (% of complete data)
Caregiver's Gender		
Male	228 (15)	66 (15)
Female	1313 (85)	365 (85)
Missing	7	1

Caregiver's Age	Mean = 41.1, SD = 7.8	Mean = 35.7, SD = 7.9

Caregiver's Marital Status		
Never Married	122 (8)	49 (11)
Married	1060 (69)	306 (71)
Living with partner	67 (4)	23 (5)
Separated or Divorced	256 (17)	46 (11)
Widowed	23 (2)	5 (1)
Missing	20	3

Caregiver's Race		
White	980 (64)	271 (64)
Black or African-American	337 (22)	94 (22)
American Indian/Alaska Native	22 (1)	3 (1)
Asian	30 (2)	10 (2)
Native Hawaiian/Pacific Is.	5 (.3)	1 (.2)
Other	107 (7)	34 (8)
Multiple Races	50 (3)	12 (3)
Missing	17	7

Caregiver's Ethnicity		
Non Hispanic	1370 (89)	381 (89)
Hispanic	167 (11)	47 (11)
Missing	11	4

Caregiver's Relationship to Child		
Mother, Stepmother, Foster Mother	1248 (81)	352 (82)
Father, Stepfather, Foster Father	211 (14)	61 (14)
Grandparent	42 (3)	11 (3)
Guardian or Other	35 (2)	2 (.5)
Missing	12	4

Caregiver's Education Level		
< = 8^th ^grade	27 (2)	3 (1)
Some high school	75 (5)	23 (5)
High school degree/GED	277 (18)	71 (17)
Some college/technical degree	529 (35)	126 (29)
College degree	433 (28)	123 (29)
Advanced degree	193 (13)	82 (19)
Missing	14	4

Child's Age (yrs)	Mean = 12.1, SD = 2.6	Mean = 6.0, SD = .83

Child's Gender		
Male	736 (48)	189 (44)
Female	809 (52)	243 (56)
Missing	3	0

Chronic Health Condition in Past 6 Months		
> = 2 Chronic Health Conditions	791 (51)	215 (50)
Most Common Chronic Health Conditions*	174 (11)	33 (8)
ADHD/ADD	91 (6)	23 (11)
Arthritis	84 (5)	18 (5)
Mental Health Conditions	54 (4)	7 (2)
GI Conditions	39 (3)	7 (2)
Diabetes	24 (2)	3 (1)
Allergies	13 (1)	9 (2)

*some participants reported more than one chronic health condition

Table 5 compares the mean KIDSCREEN-52 Parent Proxy scale scores for the PROMIS proxy sample and the KIDSCREEN normative proxy sample [28]. The two sample populations scored similarly on many scales. However, the PROMIS proxy sample scored lower on the School Environment scale and higher on the Social Acceptance and Bullying scale for both males and females. In addition, for males the PROMIS sample average was lower for Moods and Emotions, and for females the PROMIS sample average was higher for Psychological Well-Being, Parent Relations and Home Life, and Social Support & Peers.

**Table 5:** PROMIS Proxy Caregiver Mean KIDSCREEN-52 Scale Scores by Gender

Kidscreen - 52 Parent Proxy Dimension Scores* Normative Data Sample	Males		Females	
	
	N	Mean (SD)	N	Mean (SD)
Physical Well-Being	7351	51.4 (9.9)	8322	48.8 (10.0)

Psychological Well-Being	7375	50.1 (9.8)	8380	49.9 (10.2)

Moods & Emotions	7347	50.4 (10.0)	8353	49.7 (10.0)

Self Perception	7387	52.1 (9.8)	8406	48.2 (9.8)

Autonomy	7426	50.7 (9.8)	8448	49.4 (10.2)

Parent Relations & Home Life	7332	50.1 (9.8)	8354	49.9 (10.2)

Financial Resources	7282	49.8 (9.9)	8291	50.2 (10.1)

Social Support & Peers	7204	49.7 (10.2)	8261	50.2 (10.0)

School Environment	7342	49.1 (10.0)	8333	50.8 (9.9)

Social Acceptance & Bullying	7421	49.5 (10.2)	8427	50.4 (9.8)

**Kidscreen-52 Parent Proxy Dimension Scores PROMIS Sample**				

Physical Well-Being	207	51.5 (13.0)	252	49.6 (14.3)

Psychological Well-Being	207	50.9 (10.9)	248	52.1 (11.5)^a^

Moods & Emotions	208	48.1 (13.3)^b^	249	49.5 (11.7)

Self Perception	205	52.1 (11.7)	248	48.7 (11.1)

Autonomy	208	50.8 (10.0)	248	50.3 (10.7)

Parent Relations & Home Life	206	51.1 (9.8)	251	51.7 (11.0)^a^

Financial Resources	205	50.2 (10.2)	245	51.0 (10.9)

Social Support & Peers	202	50.1 (11.2)	242	52.4 (11.7)^a^

School Environment	202	51.0 (11.7)^a^	232	55.0 (11.4)^a^

Social Acceptance & Bullying	209	45.1 (13.3)^b^	246	46.8 (11.7)^b^

*KIDSCREEN proxy is to be administered in caregivers of children ages 8-18 and T scores are reported.

Normative published data [28]

^a, b^Indicates PROMIS sample means that are significantly higher (a) or lower (b) (*p *< 0.05) than the corresponding normative sample means.

Table 6 compares the PedsQL™ 4.0 Generic Core Scales Parent Proxy-Report version 4.0 mean total scores in the current PROMIS large-scale survey caregiver population to the published normative values [29] stratified by gender and age group. In general, the PROMIS proxy sample had similar scores except for 5-6 year olds. Table 7 shows a similar comparison stratified by the presence of a chronic disease diagnosis including asthma or treatment in the 6 months prior to the survey. The PROMIS sample averages for proxy reports for healthy children were higher than those for thenormative sample for all scales except Emotional Functioning, for which scores were lower. For proxy reports for children with chronic disease diagnoses, PROMIS sample averages were higher for Social Function, School Function, and the Total Score. The PROMIS asthma sample scored higher on all four PedsQL Asthma samples than the original normative sample with asthma.

**Table 6:** Mean PedsQL™ 4.0 Generic Core Scales Parent Proxy-Report Scores by Gender and Age Group

*PedsQL™Generic Core Scales*	*Total Scale Score*Mean (SD)
Normative Data Sample*-Proxy with Female Child (n = 4834)	81.5 (15.9)

Normative Data Sample *-Proxy with Male Child (n = 5236)	81.3 (15.9)

*PROMIS Sample-Proxy with Female Child (n = 256)*	82.6 (11.8)

*PROMIS Sample-Proxy with Male Child (n = 208)*	81.0 (12.1)

Normative Data Sample *-Proxy with Child Ages 5-7 years old (n = 2464)	78.0 (16.4)

Normative Data Sample *-Proxy with Child Ages 8-12 years old (n = 3152)	78.9 (16.6)

Normative Data Sample *-Proxy with Child Ages 13-1 years old (n = 1384)	79.5 (16.4)

*PROMIS Sample-Proxy with Child Ages 5-7 years old (n = 106)*	83.3 (10.8)^a^

*PROMIS Sample-Proxy with Child Ages 8-12 years old (n = 174)*	81.1 (12.1)

*PROMIS Sample-Proxy with Child Ages 8-18 years old (n = 187)*	81.9 (12.4)

*from published normative values [29]

^a^Indicates PROMIS sample means that are significantly higher (*p *< 0.05) than the corresponding normative sample means.

**Table 7:** Mean PedsQL™ 4

*PedsQL*™ *Generic Scales*	Normative Data Sample*-*Proxy with Healthy Child*	Normative Data Sample* -*Proxy with Chronic Disease Child*	*Normative Data Sample *-Proxy with Asthma Child**	*PROMIS Sample*-*Proxy with Healthy Child*	*PROMIS Sample-Proxy with Chronic Disease Child*	*PROMIS Sample-Proxy with Asthma Child*
	**Mean (SD) (n = 8713)**	**Mean (SD) (n = 831)**	**Mean (SD) (n = 145)**	**Mean (SD) (n = 289)**	**Mean (SD) (n = 177)**	**Mean (SD) (n = 379)**

Physical Functioning	84.1 (19.7)	77.0 (20.2)		89.4 (9.8)^a^	80.1 (14.3)	

Emotional Functioning	81.2 (16.4)	71.1 (19.8)		79.2 (14.7)^b^	71.3 (16.8)	

Social Functioning	83.1 (19.7)	75.1 (20.8)		89.7 (12.9)^a^	79.5 (16.6)^a^	

School Functioning	78.3 (19.6)	65.6 (21.8)		81.3 (15.2)^a^	71.2 (16.9)^a^	

Psychosocial Health	81.2 (15.3)	71.0 (17.3)		83.7 (11.7)^a^	73.2 (13.5)	

Summary						

Total Score	82.3 (15.6)	73.1 (16.5)		85.8 (10.0)	75.7 (12.1)^a^	

***PedsQL*™ *Asthma Module Scales***						

Asthma Symptoms			63.3 (21.4)			73.3 (20.4)^a^

Treatment Problems			77.3 (17.2)			81.0 (16.2)^a^

Worry			77.4 (22.4)			84.3 (19.8)

Communication			71.4 (26.9)			77.1 (26.1)

from published normative values *[29] and **[25]

^a, b ^Indicates PROMIS sample means that are significantly higher (a) or lower (b) (*p *< 0.05) than the corresponding normative sample means

## Discussion

Previously published manuscripts documents the methodology for the PROMIS pediatric self-report item banks [4]. The focus of this manuscript was to describe the PROMIS proxy-report item development process and large-scale survey that evolved from these earlier efforts. We anticipate that PROMIS items will be used widely in a variety of research settings and hence it is important to document the item development process so that findings from future research can be placed in the appropriate context. A complete list of items and the psychometric characteristics of the final PROMIS pediatric proxy-report items has been published elsewhere [24].

Proxy-report items were selected only from the PROMIS pediatric self-report items. This decision was based on the experience of our expert item development team because most researchers do not want a proxy-report item set that is different from the child self-report. In addition, all of the child-report items had undergone extensive focus groups with parents and children as well as cognitive interview testing [22,23]. It was not surprising that the cognitive interviews with proxies found no need for major item revisions to accommodate the adaption of the child self-report items to a proxy-report format. Thus, parents were involved in the item development process for the child self-report versions, and their input in the current study was not item development per se as much as cognitive debriefing on the modified items.

An interesting theme that did emerge during the cognitive interviews was that caregivers reported having a difficult time answering many of the items. This was because they did not know the information about their child either because the child did not share the information or the caregiver did not observe the child in enough settings to develop a conclusion. While this was particularly true for the social peer relationships domain, it was noted across most of the domains. This is not surprising as it has been documented that information provided by proxy respondents does not always correlate well with what is reported by the patient [8,11,13,14,16,30-32]. This suggests that the agreement between child and caregiver report is likely to vary across content areas.

The PROMIS parent proxy-report large-scale survey design described above allowed for the evaluation of the sample characteristics of the PROMIS pediatric proxy item banks. A separate article describes the psychometric characteristics of the items evaluated in this testing [24]. The final PROMIS pediatric parent proxy-report item banks were developed to provide accurate and efficient assessment of important domains of HRQOL for children including physical function (mobility 23 items; upper extremity 29 items), emotional distress (anger 5 items; depressive symptoms 14 items; anxiety 15 items), social peer relationships (15 items), fatigue (34 items), pain interference (13 items), and asthma impact (17 items).

The large-scale survey study population included caregivers of children with chronic illnesses and healthy children. This sampling strategy was designed to derive a range of representation across the latent traits. Because we envisioned item banks that measure across the continuum of the traits of interest (e.g., fatigue, physical function), it was important to include caregivers and children with a variety of experiences. In addition to allowing for broadly measured constructs, by oversampling children with asthma we were able to perform individual analyses within this disease population.

One limitation of this study was that separate analyses among specific chronic illness populations (except for asthma) was not possible due to small sample sizes for each chronic illness. In addition, due to constraints on sample size not all participants were able to be simultaneously administered both the PROMIS pediatric proxy items and the legacy scales. Finally, this study does not report on using the items in languages other than English or in other countries.

A subset of proxy respondents completed the legacy instruments. The PROMIS proxy respondents scored worse than the KIDSCREEN Normative sample on the Social Acceptance and Bullying scale. This pattern was also seen in the PROMIS child-report study [4] and may be due to cultural differences between the two populations as the PROMIS sample was based in the U.S. and the KIDSCREEN sample was European based. This would be an area for needed further research. In addition, the PROMIS proxy sample, in general, scored similar or higher on all legacy measures compared to normative samples.

The ultimate goal of the large-scale survey was to calibrate and obtain item parameters utilizing IRT which is independent of the particular sample. Hence, population diversity was more important than representativeness. This study enrolled caregivers with children who experienced a wide variety of health states (e.g., children with a variety of common chronic illnesses), age ranges, and race/ethnicity groups allowing for a diverse sample of children to be represented.

## Conclusion

This manuscript describes the process for developing the PROMIS parent proxy-report item banks and the sample for establishing item calibrations. Another paper describes the psychometric analysis leading to the final banks [24]. Further research is indicated on construct validity and tests of the responsiveness of these scales and item banks in larger samples of caregivers of pediatric patients with chronic health conditions.

## Abbreviations

PROMIS: Patient Reported Outcomes Measurement Information System; IRB: Institutional Review Board; UNC: University of North Carolina; S&W: The Children's Hospital at Scott and White in Texas; NC: North Carolina; PedsQL™ Pediatric Quality of Life Inventory™; HRQOL: health related quality of life; PRO: patient reported outcomes; UW: University of Washington CMH: Children's Memorial Hospital; LD: local dependence; DIF: differential item functioning

## Authors' contributions

DEI participated in the design of the study, interpretation of the data and drafting the manuscript. HEG participated in the data analysis, interpretation of the data and revised the manuscript. BDS participated in the data analysis, interpretation of the data and revised the manuscript. DT participated in the design of the study interpretation of the data and revised the manuscript. EMD participated in the design of the study and revised the manuscript. JSL participated in the design of the study and revising the manuscript. DA participated in the design of the study and revised the manuscript. LK participated in the acquisition of the data and revised the manuscript. JWV participated in the design of the study, interpretation of the data and revised the manuscript. DAD participated in the design of the study, interpretation of the data and drafting the manuscript. All authors read and approved the final manuscript.
